# Editorial: Plant Phytochromes: From Structure to Signaling and Beyond

**DOI:** 10.3389/fpls.2021.811379

**Published:** 2021-12-09

**Authors:** András Viczián, Cornelia Klose, Andreas Hiltbrunner, Ferenc Nagy

**Affiliations:** ^1^Biological Research Centre, Institute of Plant Biology, Laboratory of Photo- and Chronobiology, Eötvös Loránd Research Network (ELKH), Szeged, Hungary; ^2^Institute of Biology II, University of Freiburg, Freiburg im Breisgau, Germany; ^3^Centre for Biological Signalling Studies (BIOSS) and Centre for Integrative Biological Signalling Studies (CIBSS), University of Freiburg, Freiburg im Breisgau, Germany

**Keywords:** photoreceptor, phytochrome, photomorphogenesis, deetiolation, light signaling

Light is the major source of energy for plants and thus light sensing is vital for their survival. Specialized photoreceptor molecules transform the energy of the photons to chemical and biological signals that control growth and development and the adaptation to changes in the environment. Phytochromes are synthesized in their inactive (Pr) conformer and red (λ_max_ = 660 nm) light illumination can convert them to the Pfr form (Rockwell et al., 2006). Pfr is the biologically active form of phytochromes, it initiates various signaling pathways that lead to a suite of responses known as photomorphogenic development. Pfr conversion back to Pr can be triggered by far-red (λ_max_ = 730 nm) photons or by a spontaneous reversion to the lower-energy Pr state by the so-called thermal relaxation (Klose et al., 2020). There are five phytochromes (phyA-phyE) expressed in the widely used model plant *Arabidopsis thaliana* (Mathews, 2010). Since the discovery of phytochrome-mediated responses in plants (Flint, 1936; Borthwick et al., 1952) our knowledge has expanded toward multiple directions. This Frontiers Research Topic gives insights into the current state of some of these aspects of phytochrome research.

Phytochrome signaling pathways control about 10% of the Arabidopsis transcriptome (Ma et al., 2001) and these transcriptional responses are essential for environmental adaptation. Whereas, initial research efforts focused purely on examining phytochrome-dependent signaling in the context of light responses, it later became clear that phytochromes integrate light with other environmental signals such as temperature (Jung et al., 2016; Legris et al., 2019), cold stress (Franklin and Whitelam, 2007), drought stress (González et al., 2012), and pathogen attack (de Wit et al., 2013). In a review paper, Kim et al. point out that phyB, the most abundant phytochrome in light-grown plants, plays the major role in these responses. The authors summarize how phyB takes part in plant responses to diverse environmental signals and give an insight into the complexity of these signaling pathways.

Pardi and Nusinow focused on a special aspect of phytochrome signaling. It is well-established that active phytochromes translocate from the cytoplasm into the nucleus and compartmentalize into distinct molecular complexes, so-called photobodies (Yamaguchi et al., 1999; Kircher et al., 2002; Van Buskirk et al., 2012). Photobodies are essential for proper signaling but their function, composition, and biogenesis have remained unclear. Pardi and Nusinow summarized our recent knowledge about (i) possible biological roles of photobodies, (ii) protein components regulating their formation and (iii) mechanisms of photobody biogenesis including a discussion about the idea that phyB containing photobodies may be the result of liquid–liquid phase separation, similar to photobodies formed by cryptochrome 2 (Wang et al., 2021).

Although phytochromes were originally identified in seed plants (Butler et al., 1959) we now know that not only plants but also different bacteria and fungi possess phytochromes (Karniol and Vierstra, 2003). Lamparter et al. focused in their review article on *Agrobacterium* phytochromes. *Agrobacterium fabrum* contains two phytochromes called Agp1 and Agp2. The authors list the original publications leading to the discovery of Agps and summarize our recent knowledge about Agp protein structure and functions. These studies are a valuable addition to plant phytochrome research, not only from an evolutionary perspective. The advances made on *Agrobacterium* phytochrome structure determination support our efforts made on plant phytochromes, because crystallization of Agps is technically less challenging than that of plant PHYs.

Wahlgren et al. developed a methodological approach focusing on this problem. They examined the structure of recombinant Arabidopsis phyA using cryo-electron microscopy. The 17 Å resolution achieved is not exceptionally high, showing clear limitations of this approach, but the data were obtained using homodimers from near-homogenous and photochemically active protein preparations. The authors parallel their results with earlier studies performed on bacterial phytochromes and discuss further possible applications of the method.

Posttranslational modifications (PTM) occur during or after translation resulting in a covalent attachment of a moiety modifying the activity of the target protein. Phytochromes are targets of diverse PTMs; among them, phosphorylation was identified decades ago (Quail et al., 1978; Hunt and Pratt, 1980). This PTM is reversible and fine-tunes light signaling by changing the amount of available active phytochrome molecules (Stockhaus et al., 1992; Medzihradszky et al., 2013; Nito et al., 2013; Viczián et al., 2020). The two obvious regulatory steps of the phosphorylation state of the available phytochrome pools are phosphorylation and dephosphorylation. It was demonstrated that phytochromes act as serine/threonine kinases autophosphorylating themselves (Shin et al., 2016). The work of Hoang et al. added further details to the picture. The authors showed that specific missense mutations in the *Avena sativa* phyA result in increased phytochrome kinase activity. These mutant PHYAs trigger hypersensitive photoresponses when expressed in transgenic Arabidopsis plants, indicating that there is a direct positive correlation between the kinase activity of the phytochromes and the observable light responses *in planta*.

Whereas, external kinases that phosphorylate phytochromes have not been identified so far, phosphatases that dephosphorylate PHYs have already been described (Kim et al., 2002; Ryu et al., 2005; Phee et al., 2008). One of them, Phytochrome-Associated Protein Phosphatase 5 (PAPP5) dephosphorylates active Pfr phytochromes and enhances phytochrome-mediated responses (Ryu et al., 2005). von Horsten and Essen were able to crystallize recombinant PAPP5 and determine its structure at 3 Å resolution. The authors could examine the effect of different compounds on PAPP5 activity, analyze the interaction details and dynamics of PAPP5 with phospho-site mimicking mutant PHYB molecules focusing on the positional arrangement of PAPP5's known domains. Furthermore, this study indicates that the activation mode of PAPP5 is similar to that of its mammalian counterparts. The sterical consequences of PAPP5 activation by arachidonic acid suggest an exciting novel regulatory pathway linking plant defense mechanisms and phytochrome regulation by dephosphorylation, but this finding needs to be further validated in the future.

This Research Topic collected studies tackling interesting issues of the phytochrome field and rising fundamental questions that need to be addressed in the future. (i) Although the consequences of phosphorylation at certain amino acids on the function of phytochrome proteins were examined, the detailed phytochrome “phospho-map” is still missing. Furthermore, at the moment we do not know how the interplay of different PTMs fine-tune phytochrome signaling. (ii) We also do not know to what extent PHY autophosphorylation, and the activity of different kinases and phosphatases are responsible for the actual phospho-state of the available phytochrome pool and the performance of phytochrome signaling. Further targets of phytochrome kinase activity and its significance on the signaling process also need to be identified in the future. (iii) Two studies in this Research Topic focused on the molecular structure of phytochromes and their partners indicating the continued interest in the subject. Despite recent advances, we still need to achieve high resolution structural models of full length plant phytochromes in Pr and Pfr to understand the overall structural rearrangements during photoconversion. (iv) Hopefully, this structural information will allow designing structure-function models for phytochrome signaling that could be tested also experimentally. (v) Long-lasting efforts from the scientific community resulted in the identification of phytochrome interacting partners. We expect that examining the phytochrome containing photobodies will help us to understand the interaction dynamics of phytochromes with their signaling partners and to map these signaling networks in details. (vi) Furthermore, it is crucial to examine the role of phytochromes not only in light signaling but also in signal interactions of interconnecting pathways in order to understand how plants respond to complex environmental stimuli.

We, the Guest Editors of this article collection, believe that the set of papers published here stimulates further discussions and initiates studies answering the pending questions.

## Author Contributions

AV wrote the manuscript. CK, AH, and FN corrected and contributed to the text. All authors agreed in the final version.

## Funding

This work was supported by grants from the Hungarian Scientific Research Fund (OTKA, K-132633 for AV and FN and K-138022 for AV) and the German Research Foundation (DFG) under Germany's Excellence Strategy (EXC-2189—Project ID 390939984, project C1 to AH).

## Conflict of Interest

The authors declare that the research was conducted in the absence of any commercial or financial relationships that could be construed as a potential conflict of interest.

## Publisher's Note

All claims expressed in this article are solely those of the authors and do not necessarily represent those of their affiliated organizations, or those of the publisher, the editors and the reviewers. Any product that may be evaluated in this article, or claim that may be made by its manufacturer, is not guaranteed or endorsed by the publisher.
